# Complete Remission of Advanced Gastric Cancer in Response to Chemotherapy With Docetaxel, Cisplatin and Capecitabine (dcx)

**DOI:** 10.4021/wjon367w

**Published:** 2011-10-28

**Authors:** Asuncion Juarez Marroqui, Cristina Llorca Ferrandiz, Sonia Macia Escalante, Sara Blasco Molla, Dolores Garcia Ortega

**Affiliations:** a Elda General Hospital, Department of Medical Oncology. Carretera Elda-Sax, s/n. 03600. Elda (Alicante) Spain; b Elda General Hospital, Department of Radiology. Carretera Elda-Sax, s/n. 03600. Elda (Alicante) Spain

**Keywords:** Gastric cancer, Chemotherapy, Cisplatin, Docetaxel, Capecitabine

## Abstract

We describe the case of a 60 year-old man with advanced gastric cancer. The patient started chemotherapy with a combination of docetaxel, cisplatin and capecitabine. In a radiological assessment after the third treatment cycle, a significant reduction of adenopathies and gastric wall thickening was observed. Partial remission was maintained until the end of the chemotherapy (6 cycles). The follow-up evaluations indicated the radiological remission was maintained and that there was a reduction of gastric wall thickening and normalisation of remote lymph nodes. After 43 months since the final treatment cycle, the patient remains progression-free.

## Introduction

Advanced gastric adenocarcinoma is characterised by rapid growth and difficult long-term control with current chemotherapeutic treatments. The incorporation of new drugs to standard treatment regimens is leading to more and longer-lasting responses, although their use is also associated with a greater toxicity. Here, we report a case of advanced gastric cancer with an unusual development due to its remarkable response and progression-free interval.

## Case Report

A 60-year old male, ex-smoker for more than 28 years (12 pack-year history) and with a previous history of drug-treated type-2 diabetes was referred to us. The patient was admitted in the hospital in February 2007 with melena and abdominal pain. He reported a weight loss of 5 kg during the 5 months prior to admission.

**Figure 1:**
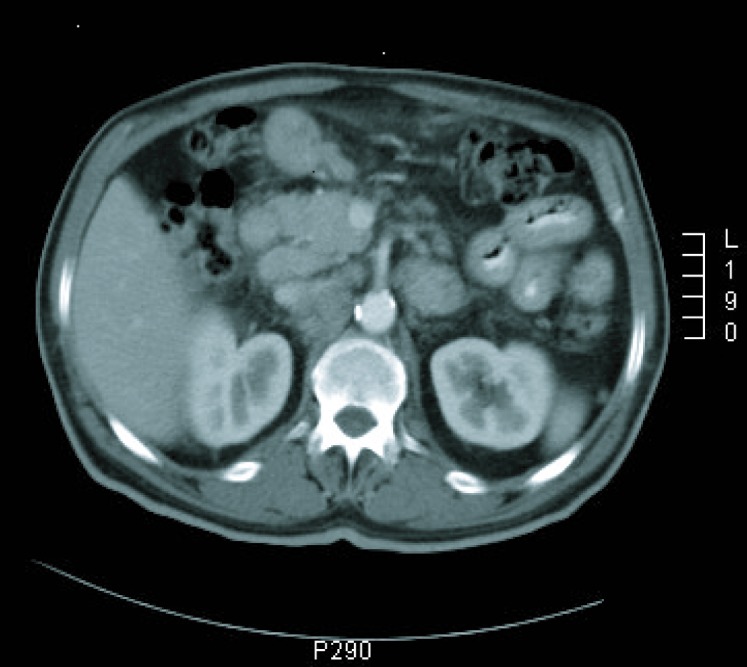
CT imaging at diagnosis.

**Figure 2:**
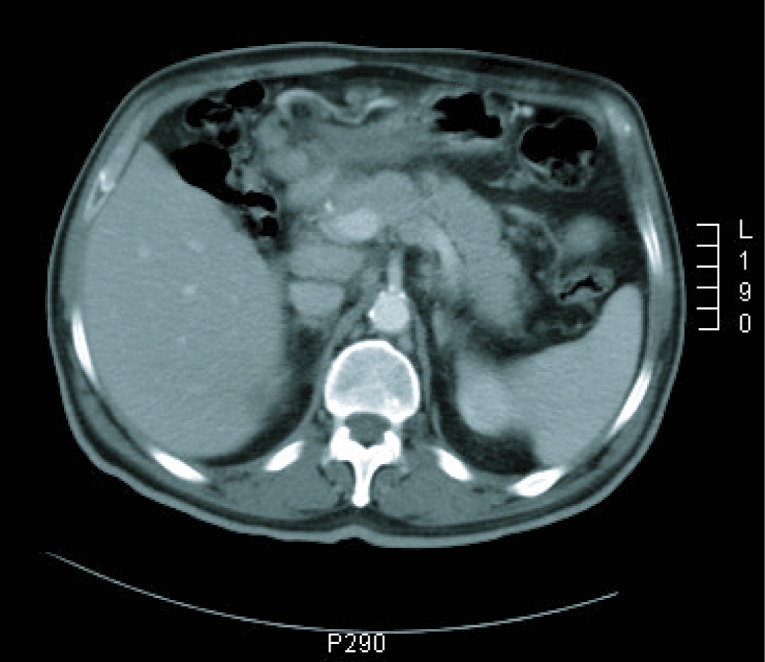
CT imaging at diagnosis.

Gastroscopy revealed a 4 cm ulcer in the gastric antrum of uncertain malignancy and a 5 mm ulcer in the body of benign appearance. Anatomical pathology analysis confirmed a diagnosis of intestinal-type gastric adenocarcinoma. It was requested an extended study with CT imaging in order to analyse gastric wall and omental thickening and a number of lymphadenopathies in the gastric, abdominal and retroperitoneal regions (Fig. 1, 2). Analysis showed that tumour markers (CEA and CA 19.9 levels) were not elevated. Given a significant lymphatic component, it was decided to perform an ultrasound-guided fine-needle aspiration (FNA) of the adenopathy, which confirmed a metastatic origin of the adenocarcinoma. Evaluated as an oncology outpatient, the subject presented a maintained general state (with an ECOG 1 performance status), with examination showing a palpable mesogastric mass.

Following a diagnosis of stage IV gastric adenocarcinoma (with lymph node metastases), it was decided to begin a chemotherapeutic treatment with docetaxel (75 mg/m^2^, day 1 every 21 days), cisplatin (60 mg/m^2^, day 1 every 21 days) and capecitabine (850 mg/m^2^, days 2-15 every 21 days), combined with granulocyte colony stimulating factors (G-CSF). Between May 2007 and August 2007, the patient received a total of 6 treatment cycles at full dosage. He only presented grade 1 toxicity, including mucositis, nausea, asthenia, hiccups, nail toxicity and neuropathy, predominantly in the first week after infusion. As haematological toxicity, the patient only presented anaemia (Grade 1) (Hg 10.6) that responded to erythropoietic stimulants.

**Figure 3:**
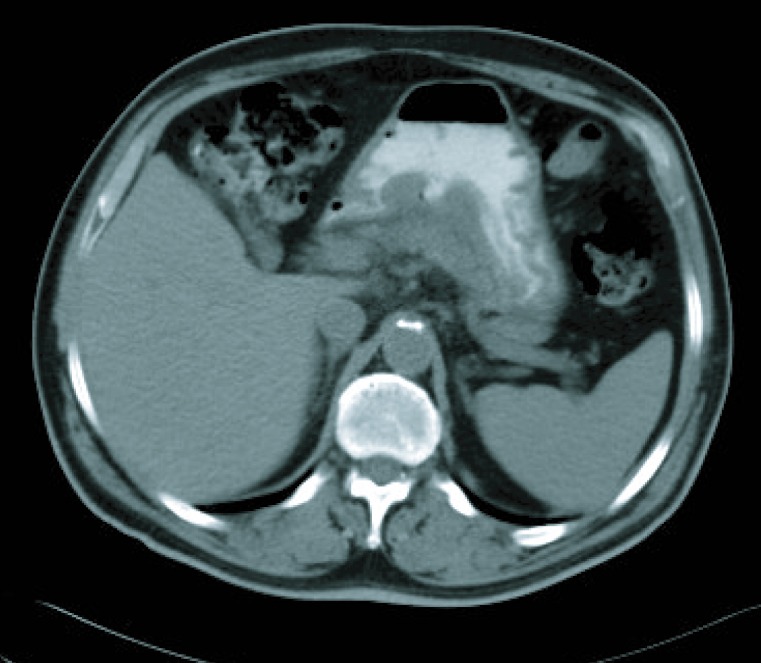
CT imaging after 6 cicles.

**Figure 4:**
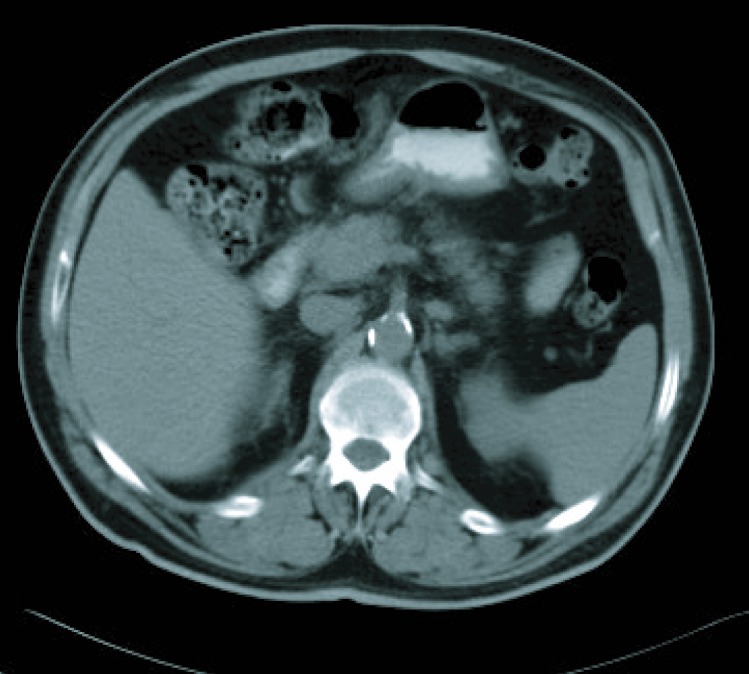
CT imaging after 6 cicles.

In a radiological re-assessment after the third treatment cycle, a significant reduction of adenopathies and gastric wall thickening was observed. Thus the response was re-evaluated as a partial remission, and after continuing for three more cycles of treatment, the radiological re-assessment maintained the same response (Fig. 3, 4). After the sixth treatment cycle was completed, the follow-up began. The patient continued with periodic evaluations, maintaining a good general state (ECOG 0) and a 14 kg weight gain since diagnosis. Follow-up CT scans showed that the radiological remission was maintained, and that there was a reduction of gastric wall thickening and normalisation of remote lymph nodes.

A follow-up gastroscopy performed in February 2010, revealed an ulcer at the angular incisures, measuring 35 mm at the maximum diameter (the ulcer was not biopsied). Following evaluation by a tumour board, and given the complete remission, a three year progression-free period, and suspected loco regional residual disease, the patient declined the offer of gastrectomy and exploratory laparotomy.

**Figure 5:**
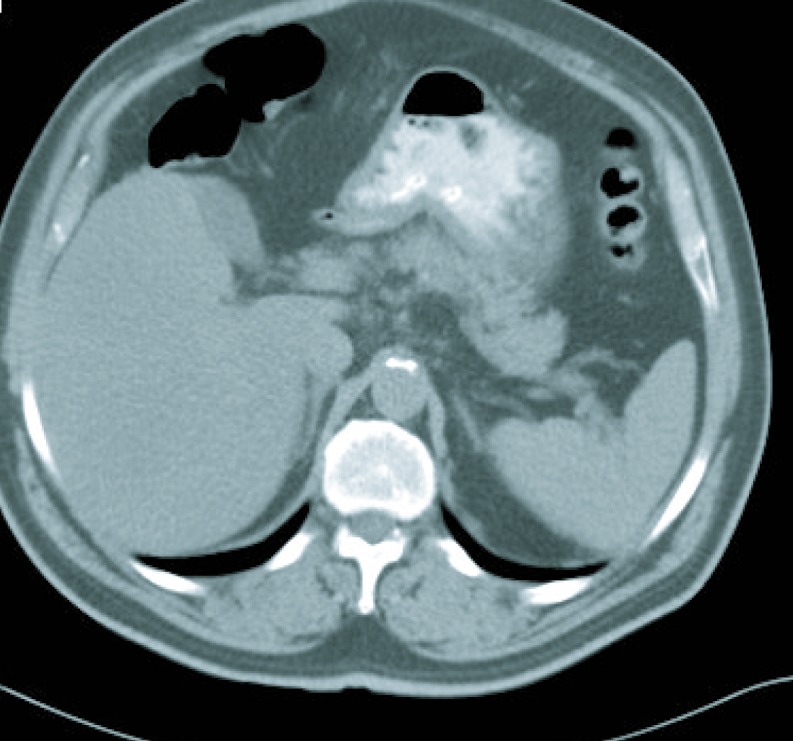
Current CT imaging.

In the last follow-up visit, in March 2011, the disease was radiologically stable (Fig. 5) and the patient was progression free for 43 months since the final treatment cycle.

## Discussion

The addition of docetaxel to standard treatment (cisplatin/5FU) has lead to a benefit in the treatment of advanced gastric carcinoma in terms of response rate and overall survival, considering the DCF scheme as a reference regimen for this pathology [1, 2]. The substitution of the 5FU infusion for oral capecitabine allows for a more comfortable administration as well as avoiding the need for central lines. Studies have shown that its replacement at different stages of gastric cancer is not detrimental in terms of progression-free survival or response rate [2, 3] and even seems to be superior. Patient selection, in terms of tolerance, is important as it is a treatment scheme with a non-negligible toxicity, although the use of G-CSF is fundamental for the control of the main haematological toxicity.

Nevertheless, taking into account that the average survival of advanced gastric cancer is 7-10 months [4], the development of the disease in the patient described was significantly satisfactory, in terms of maintaining a radiological remission, clinical benefit, and survival. It reflects the considerable heterogeneity of any neoplastic disease that is currently grouped within the same pathology, and this drives us to find the selection of patients that can most benefit from each treatment.

## References

[1] Van Cutsem E, Moiseyenko VM, Tjulandin S, Majlis A, Constenla M, Boni C, Rodrigues A (2006). Phase III study of docetaxel and cisplatin plus fluorouracil compared with cisplatin and fluorouracil as first-line therapy for advanced gastric cancer: a report of the V325 Study Group. J Clin Oncol.

[2] Cunningham D, Starling N, Rao S, Iveson T, Nicolson M, Coxon F, Middleton G (2008). Capecitabine and oxaliplatin for advanced esophagogastric cancer. N Engl J Med.

[3] Tham CK, Choo SP, Poon DY, Toh HC, Ong SY, Tan SH, Wang ML (2010). Capecitabine with radiation is an effective adjuvant therapy in gastric cancers. World J Gastroenterol.

[4] Devita V, Hellman S, Rosenberg SA (1997). Cancer principles and practice of oncology.

